# SARS-CoV-2 sero-prevalence in the workforces of three large workplaces in South Wales: a sero-epidemiological study

**DOI:** 10.1186/s12889-021-12478-x

**Published:** 2022-01-24

**Authors:** Alice Puchades, Rhian Daniel, John Geen, Jo Peden, Heather Lewis, Kelechi Nnoaham

**Affiliations:** 1 Public Health Wales, Number 2 Capital Quarter, Tyndall Street, Cardiff, CF10 4BZ UK; 2 Division of Population Medicine, Cardiff University, Neuadd Meirionnydd, Heath Park, Cardiff, CF14 4YS UK; 3 Clinical Biochemistry Department, Cwm Taf Morgannwg University Health Board, Merthyr Tydfil, CF47 9DT UK; 4 Cwm Taf Morgannwg University Health Board, Navigation Park, Unit 3 Ynysmeurig House, Abercynon, CF45 4SN UK

**Keywords:** COVID-19, SARS-CoV-2, Antibody, Sero-prevalence, Sero-epidemiology, Workplace

## Abstract

**Background:**

Sero-prevalence studies quantify the proportion of a population that has antibodies against SARS-CoV-2, and can be used to identify the extent of the COVID-19 pandemic at a population level. The aim of the study was to assess the sero-prevalence of SARS-CoV-2 antibodies in the workforce at three workplaces: a food factory, non-food factory and call-centre.

**Methods:**

Nine hundred ninety-three participants were recruited from three workplaces in South Wales. Participants completed a questionnaire and had a lateral flow point-of-care SARS-CoV-2 antibody test administered by a healthcare professional. The data were analysed using multivariable logistic regression, both using complete records only and following multiple imputation**.**

**Results:**

The sero-prevalence of SARS-CoV-2 antibodies ranged from 4% (*n* = 17/402) in the non-food factory to 10% (*n* = 28/281) in the food factory (OR 2.93; 95% CI 1.26 to 6.81). After taking account of confounding factors evidence of a difference remained (cOR comparing food factory to call centre (2.93; 95% CI 1.26 to 6.81) and non-food factory (3.99; 95% CI 1.97 to 8.08) respectively). The SARS-CoV-2 antibody prevalence also varied between roles within workplaces. People working in office based roles had a 2.23 times greater conditional odds (95% CI 1.02 to 4.87) of being positive for SARS-CoV-2 antibodies than those working on the factory floor.

**Conclusion:**

The sero-prevalence of SARS-CoV-2 antibodies varied by workplace and work role. Whilst it is not possible to state whether these differences are due to COVID-19 transmission within the workplaces, it highlights the importance of considering COVID-19 transmission in a range of workplaces and work roles.

## Introduction

Coronavirus disease (COVID-19) is an infectious respiratory disease caused by the severe acute respiratory syndrome virus 2 (SARS-CoV-2), which was declared a pandemic on 11th March 2020 [1, 2]. Serological studies, to detect the presence or absence of blood borne antibodies, help to provide a more comprehensive picture of the number of people who have previously been infected with COVID-19. They can play an important role by investigating the extent of the COVID-19 pandemic at a population level by quantifying the proportion of the population that has antibodies against SARS-CoV-2. Serological studies are particularly important to help identify COVID-19 in the population during the initial phase of the pandemic as many people were infected by COVID-19 but were not identified through antigen testing during their acute infectious period [3]. An estimated 17 to 20% of people who are infected with COVID-19 remain asymptomatic [1, 2], and limitations on COVID-19 community testing in the UK during the initial phase of the pandemic mean that widespread community testing was not available for all people with recognised symptoms of COVID-19 until 18th May 2020 [3].

There are a number of considerations for the interpretation of SARS-CoV-2 sero-epidemiological studies. Whilst antibody responses have been demonstrated post infection with SARS-CoV-2, they are not evident in the first week following infection and there is limited evidence on how long antibody titres will be maintained [4]. Asymptomatic seroconversion following exposure to SARS-CoV and SARS-CoV-2 have been documented in small cohorts; again the quality and longevity of these immunological responses are unknown [5–7]. COVID-19 sero-prevalence testing has been undertaken at a population level in countries including China [8], USA [9], Spain [10] and Switzerland [11], including the REACT-2 study in England which found that SARS-CoV-2 antibody prevalence was higher in younger adults, people from Black and South Asian ethnic backgrounds and essential workers [12]. There have also been multiple studies of COVID-19 sero-prevalence in healthcare workers [13, 14]. In Wales 89,000 people from key priority groups including health and social care workers, care home residents, teachers and pupils at education hubs underwent SARS-CoV-2 antibody testing from June–November 2020, with 11% having positive results [15]. However, this finding is not generalisable to large, enclosed workplace settings, due to the skewed demographics in healthcare and teaching settings which have workforces that are predominantly female with different ethnic backgrounds than the UK working age population [16, 17], and their different environments that involve regular close contact with members of the public.

There has also been a focus on COVID-19 transmission in a range of workplace settings. Outbreaks in meat and poultry processing plants across the UK and Europe [18], have highlighted a number of specific risk factors that explain the larger number of COVID-19 cases in these settings. These include: working environments such as low temperatures, high humidity and multiple metallic surfaces; inability to social distance; and inappropriate self-isolation linked to financial incentives to keep working despite having symptoms [19]. There have also been studies examining COVID-19 clusters in other types of workplaces including food factories, non-food factories and offices, which again have highlighted a range of risk factors for COVID-19 clusters. These include: working in confined indoor spaces; shared canteen spaces or dressing rooms; shared transport; and staff socialising in the community [20]. However, the majority of research into workplace transmission has focused on antigen testing for current COVID-19 infection, which may have missed asymptomatic individuals due to testing limitations in the initial phases of the pandemic. Understanding the sero-prevalence in workplaces adds a valuable element to the epidemiological picture in relation to past infection and potential workplace transmissions during the early phase of the COVID-19 pandemic in the UK.

## Methods

### Study design and sample

Three separate large (non-NHS) workplaces were identified within the boundaries of Cwm Taf Morgannwg University Health Board and approached to participate in the proposed sero-prevalence project. These were a food factory (not a meat processing plant), a non-food factory and a call centre. These were all in different geographical areas within Cwm Taf Morgannwg University Health Board. The ventilation and density of people varied between the different workplaces and work areas within workplaces. None of these workplaces had known outbreaks of COVID-19 that were investigated by Public Health Wales prior to their involvement in the study.

Through discussion with each Human Resources Department, agreement was obtained from the workplaces to give access to their workforce and provide suitable designated facilities for participant recruitment and sample analysis.

Each workplace was attended by health care professionals, for a 6-week period between September and October 2020. All the people working at the three workplaces were eligible to participate in the study, including those who had previously tested positive for COVID-19. Observing social distancing procedures and personal protective procedures, participants were approached and provided with a participant information sheet with a view to providing written consent to partake in the project. Once consent was provided, a whole blood capillary sample was analysed for SARS-CoV-2 antibody status following the agreed standard operating procedure. The standard operating procedure described the procedures for sampling and analysis of whole blood capillary and serum samples using the Orient Gene point-of-care lateral flow anti-SARS-CoV-2 antibody test. This document also outlined the principal of the methodology, purpose for the investigation, interpretation of the results, the appropriate handling and storage of all associated consumables and how to record the results obtained. Participants were classified as positive for anti-SARS-CoV-2 antibodies if there was an indicator line in either the IgM, IgG or both positions within the reading window.

The participant was also asked to complete a comprehensive questionnaire seeking information to help facilitate analysis of the sero-prevalence status. The questionnaire included information on participant demographics including age, sex and self-reported ethnicity, home details, occupational details including workplace and work category, mode of transport to work, symptom history and medical history including shielding status.

993 participants were recruited and the SARS-CoV-2 antibody status was recorded for subsequent statistical analysis in conjunction with the information obtained from the questionnaire. The participants were not involved in the design of this study.

### Lateral flow testing

The Orient Gene point-of-care lateral flow anti-SARS-CoV-2 antibody test (Zhejiang Orient Gene Biotech Co. Ltd., Zhejiang, China) was used to analyse capillary whole blood samples taken from consenting participants. All participants who had either IgG or IgM antibodies or both, was considered positive as having past exposure to SARS-CoV-2.

Prior to the use of the lateral flow point of care SARS-CoV-2 antibody test, a comprehensive prospective verification of the methodology was undertaken by the Cwm Taf Morgannwg University Health Board’s Clinical Biochemistry and Point of Care Testing Departments.

Clinical Sensitivity was calculated using the Orient Gene result (positive for IgM or IgG or both) and comparing against those individuals known to have had a positive SARS-CoV-2 PCR (recognised reference method for viral detection) test at least 14 days previous to sampling. Clinical Specificity was calculated using data from the serum samples archived prior to December 2019 where it was assumed all donations were from SARS-CoV-2 negative patients with no likelihood of them having had SARS-CoV-2.

The findings demonstrated a clinical sensitivity, at ≥ days 14 after either onset of symptoms or first positive SARS-CoV-2 RT-PCR result, of 97.8 and 96.0% respectively. Clinical specificity, using anonymised stored (serum) samples from September to December 2019, was 95.5%. Overall clinical accuracy was 96% and the Orient Gene lateral flow device for the detection of SARS-CoV-2 antibodies was considered suitable for clinical use.

The Orient Gene point-of-care lateral flow anti-SARS-CoV-2 antibody test was also compared against the fully automated Roche Cobas 801 SARS-CoV-2 anti-nucleocapsid antibody immuno-assay method (detects IgM and IgG antibodies to the viral nucleocapsid). The Roche immunoassay was considered the reference method for SARS-CoV-2 antibody status, for comparative purposes. The Orient Gene point-of-care lateral flow anti-SARS-CoV-2 antibody test had a Positive Percentage Agreement (PPA) of 92% and a Negative Percentage Agreement (NPA) of 97% and a concordance of 95% on comparison with the Roche immunoassay methodology for paired capillary whole blood and serum samples. Based on the evidence described above, the Orient Gene lateral flow device for the detection of SARS-CoV-2 antibodies was considered suitable for clinical use.

### Statistical analysis

Information from free text questions within the questionnaires was grouped together to create binary and categorical variables. Self-reported ethnicity was grouped into two groups, white and Black, Asian and Minority Ethnic (BAME), due to the small number of participants who reported being from BAME ethnic groups to allow for statistical analysis. Work category was split into three groups following discussions with each Human Resource Department to identify if a role was predominantly based in the office, on the factory floor or placed in the “other” category, which were made up of roles split between the office and factory floor, or based in other areas of the site. Mode of transport to work was split into two groups: those who had reported as only ever travelling to work by foot/ bicycle or in a car by themselves or with members of their own household, and those who reported ever using public transport or sharing a private vehicle with someone outside their household to travel to work.

STATA 16 was used for all statistical analyses. All variables are described (number and percentage in each category, together with histogram) first by workplace (food factory/ non-food factory/ call centre) and then by antibody test result (positive/negative). The number and percentage of missing observations for each variable is also described.

The variables included in the multivariable logistic regression model were chosen based on subject matter grounds. These were variables a priori considered to be plausibly predictive of antibody positivity that would also likely vary between workplaces. Further data-driven variable selection was not required since the number of covariates was low enough for the logistic regression model to be fitted without inducing substantial finite sample bias in the estimated coefficients.

Multiple imputation by chained eqs. (10 imputed datasets, 10 burn-in iteration per imputation) is used to address the incompleteness of the data. Each univariate imputation model contains all other variables as predictors, with the binary and categorical variables imputed using logistic and multinomial logistic regression models, respectively.

The main analysis model is a logistic regression with antibody test result as the outcome and age, sex, ethnicity, ongoing medical condition, shielding letter, household occupancy, workplace, work category and mode of transport to work as predictors. The remaining variables (symptoms of Covid-19, confirmed case in household and colleague off sick) are omitted from the main model (although they play the role of auxiliary variables in the multiple imputation) since they could be consequences, rather than causes, of the outcome.

The main analysis model is fitted to the multiply imputed datasets and the results combined using Rubin’s rules. For comparison, the results of a complete records logistic regression are also reported.

## Results

### Description of the data

Of the 993 participants recruited, 463 (47%) had all 13 relevant variables observed. Two participants were omitted due to having only age and sex observed, but the remaining 991 participants were used in the multiple imputation analysis. Table 1 shows the percentage of missing responses by variable, which ranges from 0 to 36%. Some variables such as ongoing medical condition had a higher proportion of missing data due to the question not being completed in the questionnaire, whilst 36% of ethnicity data was missing as it was not possible to categorise many of the free-text responses.

**Table 1:** Description of the data

**Variable**	**Level**	**Overall distribution**		**Distribution by workplace**						**Distribution by antibody test result**			
				Call Centre		Food Factory		Non-Food Factory		Negative		Positive	
**Age**	16-19	13	(1%)	8	(3%)	5	(2%)	0		12	(1%)	1	(2%)
	20-29	220	(22%)	98	(32%)	64	(23%)	58	(14%)	207	(22%)	13	(20%)
	30-39	250	(25%)	110	(36%)	64	(23%)	76	(19%)	232	(25%)	18	(27%)
	40-49	206	(21%)	43	(14%)	57	(20%)	103	(26%)	191	(21%)	15	(23%)
	50-59	229	(23%)	34	(11%)	57	(20%)	137	(34%)	215	(23%)	14	(21%)
	60-69	70	(7%)	10	(3%)	32	(11%)	27	(7%)	65	(7%)	5	(8%)
	*Missing*	*3 *	*(0.3%)*	*0*		*2*	*(0.7%)*	*1*	*(0.3%)*	*3*	*(0.3%)*	*0*	
**Sex**	Female	394	(40%)	166	(55%)	115	(41%)	113	(28%)	365	(39%)	29	(44%)
	Male	597	(60%)	137	(45%)	166	(59%)	289	(72%)	560	(61%)	37	(56%)
	*Missing*	*0*											
**Ethnicity**	BAME	15	(2%)	2	(1%)	4	(3%)	9	(3%)	12	(2%)	3	(7%)
	White	618	(98%)	199	(99%)	153	(97%)	263	(97%)	577	(98%)	41	(93%)
	*Missing*	*358*	*(36%)*	*102*	*(34%)*	*124*	*(44%)*	*130*	*(32%)*	*336*	*(36%)*	*22*	*(33%)*
**Ongoing medical condition**	No	634	(75%)	179	(72%)	192	(76%)	261	(77%)	589	(75%)	45	(75%)
	Yes	208	(25%)	70	(28%)	59	(24%)	77	(23%)	193	(25%)	15	(25%)
	*Missing*	*149*	*(15%)*	*54*	*(18%)*	*30*	*(11%)*	*64*	*(16%)*	*143*	*(15%)*	*6*	*(9%)*
**Shielding letter**	No	923	(97%)	288	(97%)	251	(96%)	380	(98%)	861	(97%)	62	(98%)
	Yes	27	(3%)	8	(3%)	10	(4%)	8	(2%)	26	(3%)	1	(2%)
	*Missing*	*41*	*(4%)*	*7*	*(2%)*	*20*	*(7%)*	*14*	*(3%)*	*38*	*(4%)*	*3*	*(5%)*
**No. in household (incl. self)**	1	87	(9%)	21	(8%)	27	(10%)	39	(10%)	82	(9%)	5	(8%)
	2	293	(31%)	71	(25%)	94	(35%)	126	(32%)	276	(31%)	17	(28%)
	3	230	(25%)	81	(29%)	60	(23%)	88	(22%)	214	(24%)	16	(26%)
	4	246	(26%)	82	(29%)	56	(21%)	107	(27%)	231	(26%)	15	(25%)
	5+	86	(9%)	24	(9%)	28	(11%)	34	(9%)	78	(9%)	8	(13%)
	*Missing*	*49*	*(5%)*	*24*	*(8%)*	*16*	*(6%)*	*8*	*(2%)*	*44*	*(5%)*	*5*	*(8%)*
**Covid-19 symptoms (self)**	No	720	(73%)	184	(61%)	220	(78%)	314	(78%)	685	(74%)	35	(53%)
	Yes	266	(27%)	116	(39%)	61	(22%)	86	(22%)	235	(26%)	31	(47%)
	*Missing*	*5*	*(0.5%)*	*3*	*(1%)*	*0*		*2*	*(1%)*	*5*	*(0.5%)*	*0*	
**Covid-19 case (excl. self) in household**	No	966	(98%)	294	(97%)	274	(98%)	393	(99%)	906	(99%)	60	(92%)
	Yes	18	(2%)	9	(3%)	5	(2%)	4	(1%)	13	(1%)	5	(8%)
	*Missing*	*7*	*(0.7%)*	*0*		*2*	*(1%)*	*5*	*(1%)*	*6*	*(1%)*	*1*	*(2%)*
**Workplace**	Call Centre	303	(31%)							282	(31%)	21	(32%)
	Food Factory	281	(29%)							253	(28%)	28	(42%)
	Non-Food Factory	402	(41%)							385	(42%)	17	(26%)
	*Missing*	*5*	*(0.5%)*							*5*	*(0.5%)*	*0*	
**Work category**	Factory floor	397	(40%)	0		206	(74%)	190	(47%)	376	(41%)	21	(32%)
	Office	456	(46%)	286	(95%)	27	(10%)	142	(35%)	422	(46%)	34	(52%)
	Other	133	(13%)	16	(5%)	45	(16%)	69	(17%)	123	(13%)	10	(15%)
	*Missing*	*5*	*(0.5%)*	*1*	*(0.3%)*	*3*	*(1%)*	*1*	*(0.3%)*	*4*	*(0.4%)*	*1*	*(2%)*
**Ever travel to work in shared vehicle**	No	734	(83%)	220	(84%)	174	(68%)	336	(92%)	683	(82%)	51	(91%)
	Yes	155	(17%)	41	(16%)	82	(32%)	31	(8%)	150	(18%)	5	(9%)
	*Missing*	*102*	*(10%)*	*42*	*(14%)*	*25*	*(9%)*	*35*	*(9%)*	*92*	*(10%)*	*10*	*(15%)*
**Colleague off sick with Covid-19 symptoms**	No	525	(59%)	153	(60%)	132	(51%)	237	(64%)	499	(60%)	26	(44%)
	Yes	362	(41%)	101	(40%)	126	(49%)	134	(36%)	329	(40%)	33	(56%)
	*Missing*	*104*	*(10%)*	*49*	*(16%)*	*23*	*(8%)*	*31*	*(8%)*	*97*	*(10%)*	*7*	*(11%)*
**SARS-CoV-2 antibody test result**	Negative	925	(93%)	282	(93%)	253	(90%)	385	(96%)				
	Positive	66	(7%)	21	(7%)	28	(10%)	17	(4%)				
	*Missing*	*0*											

**Fig. 1:**
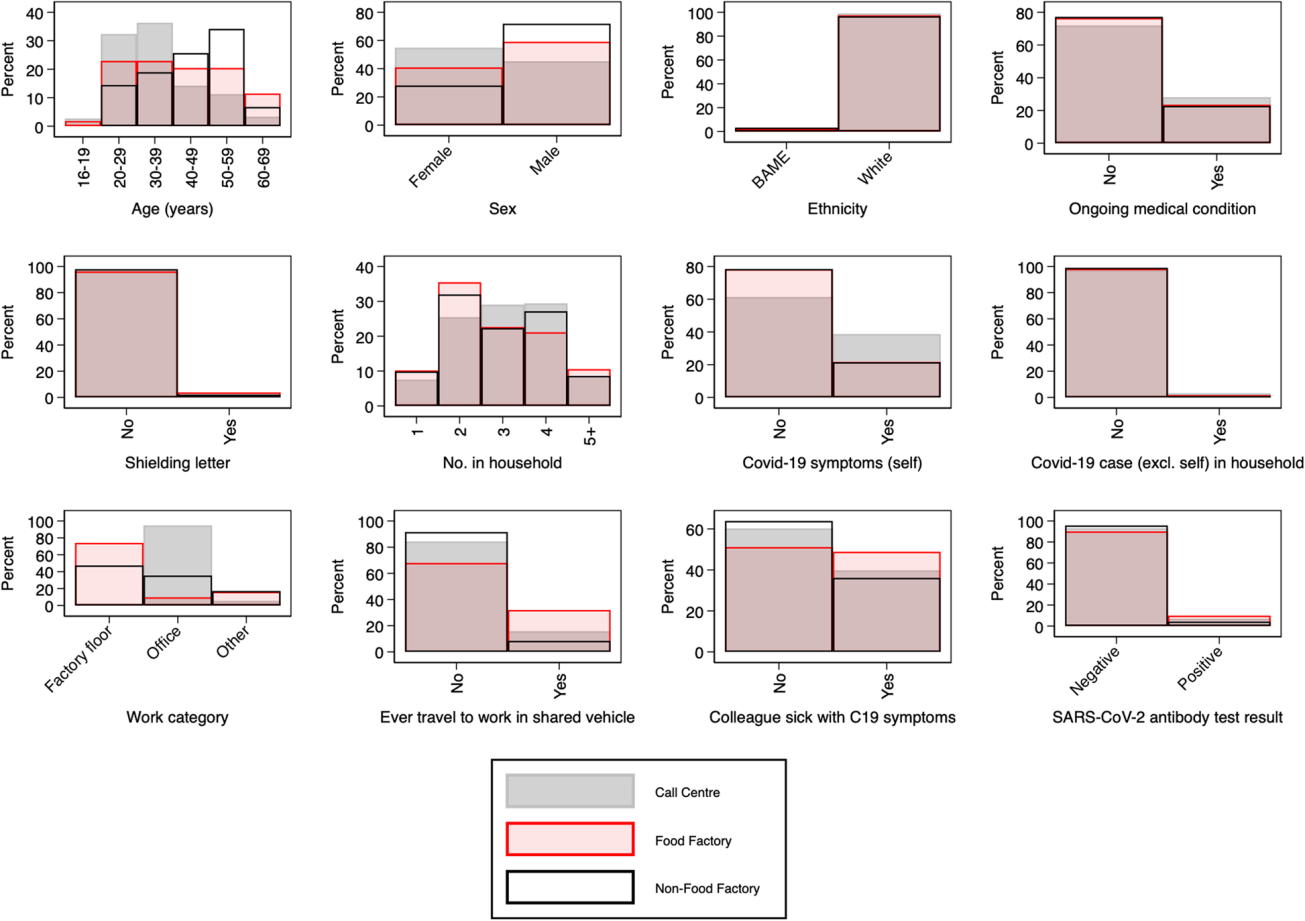
Histograms showing the distribution of each variable according to workplace

The three workplaces were approximately evenly represented in the sample, with slightly more from the non-food factory (41%, *n* = 402/991) than the other two (call centre 31%, *n* = 303/911; food factory 29%, *n* = 281/991). As shown in Table 1 (and further illustrated in Fig. 1), the distribution of age and sex varies considerably between the workplaces, with,older and more male employees at the non-food factory, and younger and more female at the call-centre. All three workplaces have workforces from predominantly white ethnic backgrounds, with workers from BAME backgrounds only accounting for between 1 and 3% of the workforce in the different workplaces. The distribution of type of job also varied with predominantly office workers in the call centre, predominantly factory floor workers in the food factory, and a mixture of factory floor, office and other locations in the non-food factory.

**Fig. 2:**
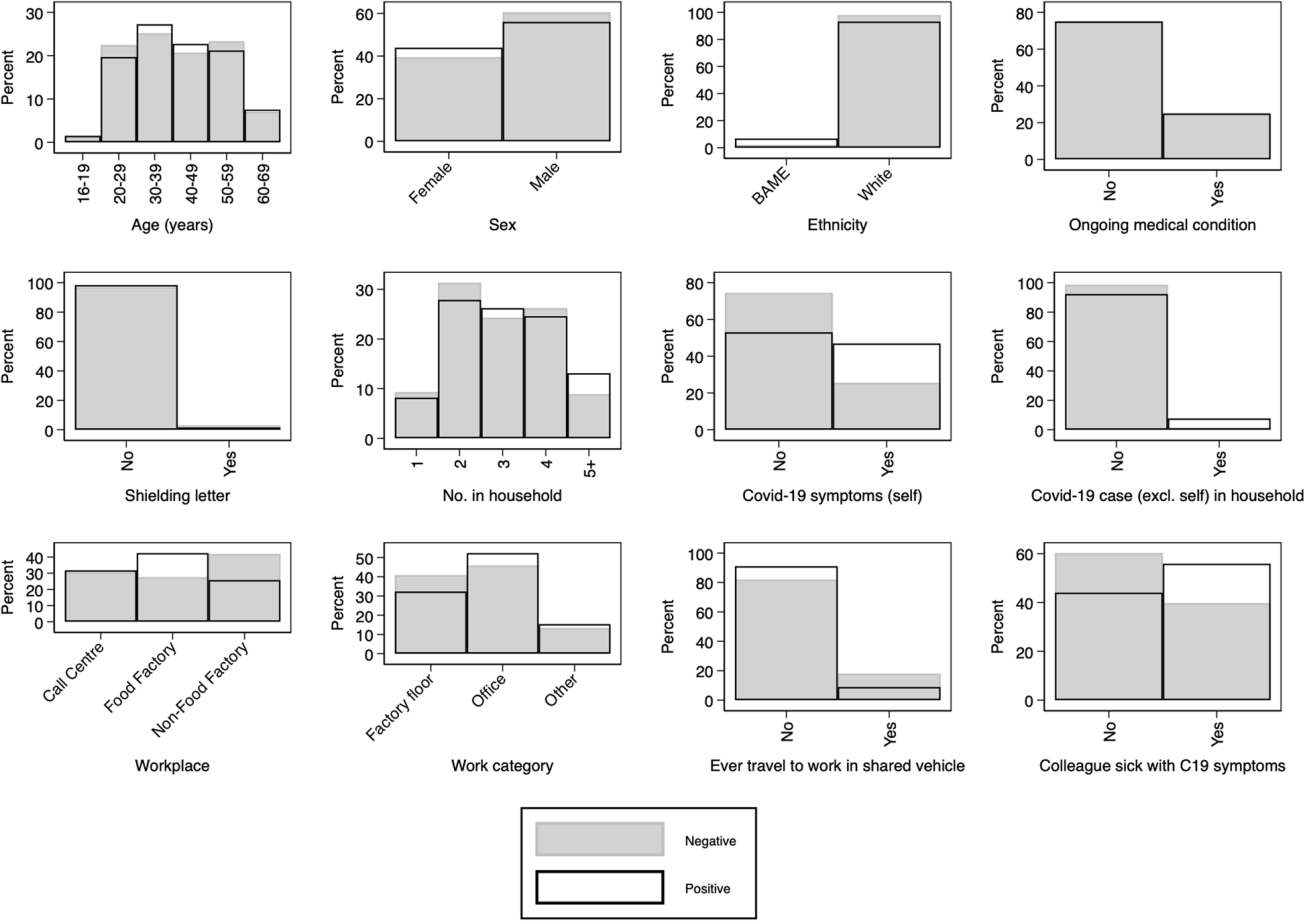
Histograms showing the distribution of each variable according to antibody positivity

Overall, 7% (*n* = 66/991) tested positive for antibodies, a prevalence that ranged from 4% (*n* = 17/402) in the non-food factory to 10% (*n* = 28/281) in the food factory. Out of the 66 people who tested positive for antibodies 3 tested positive for IgM alone suggesting a very recent infection, 37 tested positive for IgG alone indicating a historic infection, and 26 tested positive for both IgG and IgM indicating a recent infection where the antibody isotype is switching from an IgM to an IgG isotype. Table 1 (and Fig. 2) also show an association between having experienced symptoms of Covid-19 and antibody positivity, although over half (54%, *n* = 35/66) of those testing positive for antibodies had not reported experiencing symptoms.

### Logistic regression

Table 2 shows the results of the logistic regression analyses, both that conducted on the complete records alone, and those conducted (and subsequently combined) on the ten multiply imputed datasets.

**Table 2:** Results of the multivariable logistic regression analysis (outcome: antibody positivity, predictors as shown above) on both the complete records and multiply imputed data

**Variable**	**Level**	**Complete Records **(n=463)			**Multiple Imputation **(n=991)		
		Estimated conditional OR	95% CI	p-value	Estimated conditional OR	95% CI	p-value
**Age**	16-19	1			1		
	20-29	0.34	0.03–3.80		0.94	0.11–8.39	
	30-39	0.43	0.04–4.46		1.10	0.13–9.63	
	40-49	0.86	0.08–9.15	0.36	1.25	0.14–11.1	0.97
	50-59	0.97	0.09–10.5		1.19	0.13–10.8	
	60-69	0.87	0.06–13.3		1.04	0.10–10.7	
**Sex**	Female	1			1		
	Male	1.34	0.59–3.05	0.49	0.88	0.51–1.51	0.64
**Ethnicity**	BAME	1			1		
	White	0.23	0.05–0.96	0.04	0.39	0.11–1.39	0.15
**Ongoing medical condition**	No	1			1		
	Yes	1.18	0.51–2.75	0.70	1.12	0.58–2.16	0.74
**Shielding letter**	No	1			1		
	Yes	0.89	0.11–7.42	0.91	0.35	0.04–2.84	0.33
**No. in household (incl. self)**	1	1			1		
	2	0.67	0.15–2.91	0.71	1.03	0.35–2.99	0.75
	3	1.04	0.24–4.42		1.12	0.38–3.29	
	4	1.43	0.36–5.66		1.02	0.35–2.97	
	5+	1.04	0.18–6.03		1.82	0.54–6.11	
**Workplace**	Call Centre	1			1		
	Food Factory	1.07	0.34–3.41	0.08	2.93	1.26–6.81	<0.01
	Non-Food Factory	0.39	0.13–1.14		0.73	0.33–1.60	
**Work category**	Factory floor	1			1		
	Office	2.00	0.66–6.11	0.40	2.23	1.02–4.87	0.11
	Other	1.97	0.60–6.51		1.81	0.78–4.19	
**Ever travel to work in shared vehicle**	No	1			1		
	Yes	0.18	0.02–1.50	0.11	0.37	0.14–0.95	0.04
**Baseline odds**		0.40	0.02–10.0		0.08	0.005–1.33	

We note that the average relative increase in variance (RVI) for the coefficients of the logistic regression model due to the missing data is estimated to be 6%. That is, under the assumption that the missing data are Missing at Random (MAR) and that the imputation models are correctly specified, the amount of missing information is small, suggesting that multiple imputation is a sensible approach.

We find little evidence of an association between either age or sex with antibody positivity, after conditioning on all other variables in the model. However, we note that the confidence intervals are wide particularly for the conditional ORs comparing different age categories. There is very weak evidence (*p* = 0.15) that those from BAME backgrounds are more likely (estimated conditional OR 1/0.39 = 2.6) to be antibody positive, but this estimate is based on very low numbers of participants.

There is little evidence of a conditional association between having an ongoing medical condition and test positivity. Those who reported having received a shielding letter appear to have a slightly reduced risk of infection, although again this is not statistically significant and is based on a very low number (3%) in receipt of a shielding letter.

Although there is some suggestion that those in households with five or more occupants have a higher risk of antibody positivity, the conditional association between household size and the outcome is not statistically significant (*p* = 0.75).

There is strong evidence (*p* < 0.01) of a conditional association between the workplace and antibody positivity, conditional on all other variables in the model, with a higher infection prevalence in the food factory (estimated conditional ORs 2.93 (95% CI 1.26 to 6.81) and 3.99 (95% CI 1.97 to 8.08) relative to the call centre and non-food factory respectively). Note that this result was masked in the complete records analysis, in part due to a large proportion (18 of the 28) of the antibody positive participants from the food factory having missing data on one or more of the other variables.

It is important to note that the higher prevalence of infection seen in the food factory is seen even though work category, which might otherwise be used as a possible explanation, is included in the model. Indeed, the association with work category is perhaps somewhat surprising, with office work estimated to have 2.23 (95% CI 1.02 to 4.87) times higher conditional odds of infection compared with the factory floor.

Finally, perhaps the most surprising result is that on the mode of transport to work, comparing those who have ever travelled to work in a shared vehicle compared to those who have never travelled to work in a shared vehicle. The crude comparison of antibody positivity between these two groups, along with the adjusted comparison in both the complete records and multiple imputation analyses suggest that infection is lower amongst the group that has ever travelled in a shared vehicle to work, which is surprising as sharing a vehicle to work is a potential route of COVID-19 transmission.

## Discussion

The sero-prevalence of SARS-CoV-2 antibodies was different between the workforces of three large workplaces in Wales following lateral flow antibody testing undertaken in September and October 2020, ranging from 4% (*n* = 17/402) in the non-food factory to 10% (*n* = 28/281) in the food factory. Even after taking into account differences between these workplaces in a range of factors including age, sex, and work category, evidence of a difference remained which indicates that the prevalence of COVID-19 varied between the workforces during the first wave of COVID-19 in the UK from February–September 2020. There were also differences in SARS-CoV-2 antibody prevalence between roles within workplaces, with those working in office based roles having a 2.23 times greater conditional odds of being positive for SARS-CoV-2 antibodies than those working on the factory floor.

It is not possible to infer whether these differences in prevalence of SARS-CoV-2 antibodies, both between and within workplaces, are due to transmission of COVID-19 in the workplaces themselves. None of these workplaces had outbreaks of COVID-19 reported to Public Health Wales prior to their involvement in this study. The higher prevalence of COVID-19 in the food factory is not surprising given the multiple COVID-19 outbreaks and favourable conditions for SARS-CoV-2 transmission identified in meat and poultry facilities which share many of the same characteristics of other food factories [18, 19]. The higher prevalence in office workers compared to factory floor workers aligns with the ECDC findings that there have been COVID-19 outbreaks and clusters in a wide range of workplaces [20], and highlights the importance of focussing on potential COVID-19 transmission in a range of work settings.

Only 47% (*n* = 31/66) of people who were SARS-CoV-2 antibody positive reported having had one of the three main COVID-19 symptoms since February 2020, with the remaining 53% (*n* = 35/66) not reporting symptoms. This compares to a reported 17 to 20% of people who have asymptomatic COVID-19 infections [1, 2]. The higher rate of reported asymptomatic infections in this study could be due to recall bias, as people were completing this questionnaire around six months after the start of the COVID-19 pandemic and therefore may not accurately recall historic symptoms. However, it could also be due to people not wanting to admit to previous symptoms, due to the implications around self-isolating. It is estimated that only 18 to 25% of people with COVID-19 symptoms in the UK adheres to self-isolation fully due to the financial implications, lack of understanding on the need to self-isolate, and effects on psychological wellbeing [16]. The percentage of asymptomatic infections also varied between the workplaces, with 2% (*n* = 8/400) of people having positive SAR-CoV-2 antibodies but not reporting COVID-19 symptoms in the non-food factory, 3% (*n* = 9/300) in the call centre and 6.4% (*n* = 18/281) in the food factory. This is compared to 2.3% (n = 9/400) of people having SARS-CoV-2 antibodies and reporting COVID-19 in the non-food factory, 4% (*n* = 12/300) in the call centre and 3.6% (*n* = 10/281) in the food factory. This may indicate that workers in the food factory were less likely to report COVID-19 symptoms than people who worked in the non-food factory and call centre, potentially due to financial or psychological concerns over COVID-19 isolation.

The logistic regression included multiple potential confounding factors including demographic, medical, household and travel variables. However, there may have been residual uncontrolled confounding due to either additional confounding factors that were not considered or inaccurate reporting and categorisation of those confounding factors that were measured, which may explain the differences in SARS-CoV-2 prevalence between workplaces and work roles. For example, the grouping of roles into factory floor, office and other leaves potentially large differences remaining between the workplaces in the exact nature of the roles performed within these broad categories. The differences may also be reflective of trends of COVID-19 infection in the wider community, as the three workplaces are dispersed geographically across the Cwm Taf Morgannwg University Health Board area. However, as COVID-19 antigen testing was not available for all symptomatic individuals in the UK until 18th May 2020 [3], accurate data on the true level of COVID-19 infection in the community in the first wave of the pandemic is not available.

This study found little evidence of differences in SARS-CoV-2 antibody prevalence by age, sex, ongoing medical condition, shielding status, or number of people in the household. However, there may therefore be differences in risk of COVID-19 infection not detected in this study due to the relatively low prevalence and small sample size.

The one demographic characteristic that was found to be related to the prevalence of SARS-CoV-2 antibodies after conditioning on the other factors in the model was ethnicity. For the complete records analysis people from white ethnic backgrounds had statistically significant lower odds of having SARS-CoV-2 antibodies than those from BAME backgrounds however this was not statistically significant in the multiple imputation analysis This is in line with findings from the REACT-2 study which found that people from Black or South Asian ethnicities had 2–3 times higher prevalence of SARS-CoV-2 antibodies than people from White ethnicities [12]. However, only 15 participants (2%) in this study were from BAME backgrounds, with missing ethnicity data for 358 (36%) of participants. This may be a true reflection of the ethnic background of the participants, as the proportion of people from BAME backgrounds living in the Local Authorities within the Cwm Taf Morgannwg University Health Board area, is estimated at between 2.3 to 3.4% [17]. However, ethnicity was a free-text question in the participants’ questionnaire, and many of the responses could not be categorised. A clearer link between ethnicity and sero-prevalence may be seen if a similar study was carried out in an area with a higher proportion of participants from BAME backgrounds, or if the ethnicity question was adapted to multiple choice.

Unexpectedly people who had ever used a shared vehicle as transport to work, both public transport or a private car shared with someone outside their household, had a lower prevalence of SARS-CoV-2 antibodies than those who had never shared a vehicle with others to travel to work This is counter-intuitive as being in an enclosed space within a shared vehicle would allow for transmission of COVID-19, and previous studies have demonstrated the potential for transmission of airborne infections within vehicles [21]. Welsh Government COVID-19 guidance on travel recognised this potential for transmission within shared vehicle and provides advice on reducing possible transmission whilst using public transport, and did not recommend car sharing with people from outside an individual’s extended household at the time of the study [22]. It is possible that there is an unknown cofounding factor, which has not been considered in this study, which is causing this relationship between travelling in a shared vehicle and COVID-19 infection. However, we believe it unlikely that this finding is due to uncontrolled confounding by deprivation. People who never share a vehicle are more likely to have access to a private vehicle, and are therefore probably more affluent than those who travel to work by public transport or car share.

### Strengths and weaknesses of the study

This study adds to the evidence base as it highlights the link between workplace and work role, and prevalence of SARS-CoV-2 antibodies. It demonstrates the need to consider COVID-19 transmission in a range of workplace settings, including offices, rather than focussing solely on workplaces that are perceived as high risk of COVID-19 transmission, such as factories. This is particularly important for countries, including the UK, to consider as they examine how to manage COVID-19 in workplaces and how they ease COVID-19 restrictions. However, it should be noted that the analysis of SARS-CoV-2 prevalence based on work role is largely based on extrapolation. There were no factory workers in the call centre, and very few office workers in the food factory, the majority of the direct information on comparing work category within workplace comes from the non-food factory.

The study also adds to our understanding of asymptomatic COVID-19 infections.. As widespread community testing was not available for all symptomatic individuals prior to mid-May [3] there is limited data on and therefore an underestimation of the true prevalence of COVID-19 in the community in the UK during the first wave of the COVID-19 pandemic. This sero-prevalence study was therefore able to provide an insight into the prevalence of COVID-19 in the population during the first wave of COVID-19.

The conditional associations between demographic, health, work, home and travel risk factors should only very cautiously be given a causal inference due to the possibility of unmeasured confounding by other factors. It is known that there are other risk factors for SARS-CoV-2 infection which were not measured in this study including socioeconomic status and household income [12]. The presence of unmeasured confounding factors may explain the unexpected finding that people who never travel in a shared vehicle have higher prevalence of SARS-CoV-2 antibodies than those who travelled in shared vehicles.

The questionnaire design could have been improved by changing ethnicity and job role from free-text to multiple choice questions to allow for more accurate and complete categorisation. Many ethnicity responses could not be categorised into an ethnic group, for example anyone who responded “British” was classified as missing, as it was not possible to infer ethnicity from this response. There were hundreds of variations of job titles included in the questionnaire, and it required extensive discussions with the respective HR departments to correctly categorise these roles.

A number of assumptions were made in the statistical analyses. All the statistical inferences in this study are based on an assumption that individual outcomes (antibody positivity) are conditionally independent given the nine variables included in the model. This assumption is not true for an infectious disease studied within populations that could plausibly be infecting one another. As a result, the reported confidence intervals and *p*-values are too narrow and too small, respectively, since they don’t allow for the inherent statistical dependence between participants. The analyses also used an MAR assumption for the missing data, and parametric assumptions for the imputation model. Even with the above concerns aside, additional care must be taken when interpreting the estimated conditional ORs from the logistic regression model results in Table 2, to avoid the so-called Table 2 Fallacy [23].

## Conclusions

We found evidence of differences by workplace and work role in the sero-prevalence of SARS-CoV-2 antibodies amongst employees at three large workplaces in Wales, indicating differing infection rates for COVID-19 during the first wave of the pandemic in the UK. Employees who worked in the food factory had a statistically significant higher prevalence of SARS-CoV-2 than those who worked in the non-food factory, whilst employees who worked in predominantly office based roles had a small, but statistically significant, increased prevalence of SAR-CoV-2 compared to those who worked predominantly on the factory floor. Whilst it is not possible to state whether these differences were due to COVID-19 transmission within the workplaces, this study highlights the importance of considering the potential for COVID-19 transmission in a range of workplaces and work settings. This is particularly important for countries to consider as they examine how to manage COVID-19 restrictions in a wide range of workplaces.

## Data Availability

The datasets used during the current study are available from the corresponding author on reasonable request following approval of the Cwm Taf Morgannwg Research and Development Committee.

## Abbreviations

BAME: Black, Asian and Minority Ethnic
COVID-19: Coronavirus Disease 2019
ECDC: European Centre for Disease Prevention and Control
HR: Human Resources
IgG: Immunoglobulin G
IgM: Immunoglobulin M
MAR: Missing at Random
NHS: National Health Service
OR: Odds Ratio
RT-PCR: Reverse Transcription Polymerase Chain Reaction
RVI: Relative Increase in Variance
SARS-CoV-2: Severe Acute Respiratory Syndrome Virus 2

## References

[1] Garcia D, Egli-Gany D, Counotte MJ (2020). Occurrence and transmission potential of asymptomatic and presymptomatic SARS-CoV-2 infections: a living systematic review and meta-analysis. PLoS Med.

[2] Byambasuren O, Cardona M, Bell K, Clark J, McLaws M-L, Glasziou P (2020). Estimating the extent of asymptomatic COVID-19 and its potential for community transmission: systematic review and meta-analysis. J Assoc Med Microbiol Infect Dis Can.

[3] Everyone in the United Kingdom with symptoms now eligible for coronavirus tests. 18 May 2020. https://www.gov.uk/government/news/everyone-in-the-united-kingdom-with-symptoms-now-eligible-for-coronavirus-tests?utm_source=932565f9-f9d7-45ec-b964-d9f353f71948&utm_medium=email&utm_campaign=govuk-notifications&utm_content=daily

[4] Deeks JJ, Dinnes J, Takwoingi Y (2020). Cochrane COVID-19 Diagnostic Test Accuracy Group. Antibody tests for identification of current and past infection with SARS-CoV-2[PubMed]. Cochrane Database Syst Rev.

[5] Shields A, Faustini SE, Perez-Toledo M (2020). SARS-CoV-2 seroprevalence and asymptomatic viral carriage in healthcare workers: a cross-sectional study. Thorax..

[6] Hains DS, Schwaderer AL, Carroll AE, et al. Asymptomatic seroconversion of immunoglobulins to SARS-CoV-2 in a pediatric dialysis unit. JAMA. 2020;323:2424–5. 10.1001/jama.2020.8438 PMC7226282 32407440

[7] Chen WQ, Lu CY, Wong TW (2020). Anti–SARS-CoV immunoglobulin G in healthcare workers, Guangzhou, China. Emerg Infect Dis.

[8] Xu X, Sun J, Nie S (2020). Seroprevalence of immunoglobulin M and G antibodies against SARS-CoV-2 in China. Nat Med.

[9] Sood N, Simon P, Ebner P (2020). Seroprevalence of SARS-CoV-2–specific antibodies among adults in Los Angeles County, California, on April 10-11, 2020. JAMA..

[10] Pollán M, Pérez-Gómez B, Pastor-Barriuso R (2020). Prevalence of SARS-CoV-2 in Spain (ENE-COVID): a nationwide, population-based seroepidemiological study. Lancet.

[11] Stringhini S, Wisniak A, Piumatti G (2020). Seroprevalence of anti-SARS-CoV-2 IgG antibodies in Geneva, Switzerland (SEROCoV-POP): a population-based study. Lancet.

[12] Ward H, Atchison C, Whitaker M (2021). SARS-CoV-2 antibody prevalence in England following the first peak of the pandemic. Nat Commun.

[13] Grant J, Wilmore S, McCann N (2021). Seroprevalence of SARS-CoV-2 antibodies in healthcare workers at a London NHS trust. Infect Control Hosp Epidemiol.

[14] Garcia-Basteiro AL, Moncunill G, Tortajada M (2020). Seroprevalence of antibodies against SARS-CoV-2 among health care workers in a large Spanish reference hospital. Nat Commun.

[15] Testing Strategy for Wales. 2021. https://gov.wales/sites/default/files/publications/2021-01/testing-strategy-for-wales.pdf

[16] Scientific Pandemic Influenza Group on Behaviours. The impact of financial and other targeted support on rates of self-isolation or quarantine. 16^th^ September 2020. https://www.gov.uk/government/publications/spi-b-impact-of-financial-and-other-targeted-support-on-rates-of-self-isolation-or-quarantine-16-september-2020

[17] StatsWales. Ethnicity by area and ethnic group. 2021. https://statswales.gov.wales/Catalogue/Equality-and-Diversity/Ethnicity/ethnicity-by-area-ethnicgroup

[18] Middleton J, Reintjes R, Lopes H (2020). Meat plants—a new front line in the covid-19 pandemic. BMJ..

[19] Durad-Moreau Q, Adisesh A, Mackenzie G, et al. COVID-19 in meat and poultry facilities: a rapid review and lay media analysis. *Centre for*. Evidence-Based Medicine. 2020; https://www.cebm.net/covid-19/what-explains-the-high-rate-of-sars-cov-2-transmission-in-meat-and-poultry-facilities-2/.

[20] ECDC (2020). COVID-19 clusters and outbreaks in occupational settings in the EU/EEA and the UK.

[21] Nibbs L, Morawska L, Bell S (2012). The risk of airborne influenza transmission in passenger cars. Epidemiol Infect.

[22] Welsh Government (2021). Travelling safely during the coronavirus pandemic: alert level 4 guidance for the public.

[23] Westreich D, Greenland S (2013). The table 2 fallacy: presenting and interpreting confounder and modifier coefficients. Am J Epidemiol.

